# Neighborhood Characteristics Related to Changes in Anthropometrics During a Lifestyle Intervention for Persons with Obesity

**DOI:** 10.1007/s12529-024-10317-y

**Published:** 2024-09-11

**Authors:** Boëlle J. Brouwer, Susanne Kuckuck, Renate E. H. Meeusen, Mostafa Mohseni, Robin Lengton, Frank J. van Lenthe, Elisabeth F. C. van Rossum

**Affiliations:** 1 Department of Internal Medicine, Division of Endocrinology, Obesity Center CGG, Erasmus MC, University Medical Center Rotterdam, Rotterdam, The Netherlands; 2 Department of Public Health, Erasmus MC, University Medical Center Rotterdam, Rotterdam, The Netherlands

**Keywords:** Lifestyle intervention, Obesity, Neighborhood characteristics, Anthropometrics

## Abstract

**Background:**

Since obesity has emerged as a major public health concern, there is an urgent need to better understand factors related to weight gain and treatment success.

**Methods:**

This study included 118 persons with obesity who participated in a multidisciplinary combined lifestyle intervention with cognitive-behavioral therapy at the outpatient clinic of the Obesity Center CGG at Erasmus University Medical Center, Rotterdam, The Netherlands. Neighborhood characteristics were assessed using a 13-item questionnaire. Multiple regression analyses were performed to examine the association between perceived safety, social cohesion, and the availability of facilities on relative changes in body mass index and waist circumference changes, adjusted for corresponding neighborhood socioeconomic status scores.

**Results:**

Higher total scores, indicating more unfavorable neighborhood perceptions, were associated with less relative improvements in BMI and waist circumference after 1.5 years (*β* = 3.2, 95%CI 0.3–6.0; *β* = 3.4, 95%CI 0.3–6.6, respectively). Also, more neighborhood unsafety was associated with less relative improvements in BMI and waist circumference on the long term (*β* = 3.1, 95%CI 1.1–5.1; *β* = 2.8, 95%CI 0.6–5.1, respectively).

**Conclusion:**

The results indicate that living in a neighborhood perceived as less favorable may lower the chances of successful weight loss in response to combined lifestyle interventions in persons with obesity.

**Supplementary Information:**

The online version contains supplementary material available at 10.1007/s12529-024-10317-y.

## Introduction

Overweight (body mass index (BMI) 25 to 30 kg/m^2^) and obesity (BMI ≥ 30 kg/m^2^) have emerged as major public health concerns, both nationally and internationally, with their prevalence increasing over the past few decades [1–4]. Thus, there is an urgent need to gain a better understanding of factors associated with weight gain and treatment success.

Numerous interventions have been developed to address the escalating rates of overweight and obesity [2]. These approaches aim to control obesity with, for example, lifestyle interventions, pharmacological treatment [5, 6], and bariatric surgery [7]. As lifestyle interventions are the cornerstone of most obesity treatments, this study specifically focuses on this type of intervention. Lifestyle interventions are often used for obesity treatment, encompassing a combination of dietary interventions, physical activity interventions, and behavioral modifications [7, 8]. Several studies have evaluated the effectiveness of lifestyle interventions in diverse populations. Overall, lifestyle interventions have shown beneficial effects in reducing body weight and improving related health outcomes [9]. However, it is worth noting that the magnitude of the effects observed in the literature varies [10–12]. This variability could potentially be attributed, at least partly, to the broader social circumstances in which people carry out these interventions.

One of these social circumstances is the influence of an individuals’ neighborhood [13]. It is known that neighborhood characteristics can influence various aspects of peoples’ healthy lifestyles [14–23]. Differences in overweight rates across neighborhoods suggest that the environment might also play a role in an individual’s risk of overweight or obesity [24–26].

Indeed, numerous studies have demonstrated that individuals’ weight status can be influenced by various factors associated with neighborhood characteristics, including the built environment, social environment, and neighborhood deprivation [27–31]. Results from previous studies for example showed that people walked more if they lived in more walkable communities [30] and that people spend more time outdoors in neighborhoods with more green space, with positive impact on BMI via increased physical activity and even reduced stress levels [32]. Studies highlight various mechanisms linking neighborhood characteristics to obesity and healthy behavior. Positive social environments are associated with a lower prevalence of obesity and an increased odds of sufficient physical activity [14, 33], and the presence of healthy food stores near schools and households is associated with lower risks of obesity [22, 34]. Furthermore, green spaces promote physical activity, diminishing the risk of overweight and obesity [23, 35]. Communities with more access to physical activity facilities show higher physical activity levels [36] and research indicates that the lower the ratio of fast-food restaurants and convenience stores to grocery stores and produce vendors around people’s homes, the lower the odds of living with obesity [37]. Safety concerns, such as exposure to high levels of neighborhood crime, seem another mechanism in which the neighborhood influences obesity [16, 38]. An increase in neighborhood crime rates is associated with an increase in BMI and the likelihood of being obese [38]. This mechanism can be explained by the fact that residing in a neighborhood with high levels of violent crime is linked to less physical activity [36, 38, 39]. Besides the mechanisms described above, studies show that disadvantaged neighborhoods tend to have a higher prevalence of obesity, and they find an association between neighborhood socioeconomic status and obesity-related unhealthy behaviors [15, 40].

If neighborhood characteristics increase the probability of becoming overweight or obese [15, 32, 41–45], they may also affect the effectiveness of lifestyle interventions. Yet, there remains a limited understanding of the role of neighborhood factors in relation to the impact of lifestyle interventions on overweight and obesity [15, 17, 46]. Multidisciplinary combined lifestyle interventions (CLIs) integrate the essential components of dietary advice, physical activity, and psychological support to promote behavioral change [8, 47, 48]. Apart from a few local studies [17], it remains unclear whether the effectiveness of CLIs depends on the environment in which individuals reside.

This study examines the associations of specific self-reported neighborhood characteristics with changes in waist circumference and BMI in response to a multidisciplinary 1.5-year CLI for individuals with obesity. We hypothesized that weight loss in people living with obesity, within the context of a lifestyle intervention, is more effectively facilitated in neighborhoods with perceived fewer safety concerns, greater neighborhood attractiveness, and a lower density of fast-food outlets. Thus, we hypothesized that, individuals with obesity living in self-perceived less favorable neighborhoods may have greater challenges regarding BMI and waist circumference (WC) reduction following a combined lifestyle intervention.

## Methods

### Participants

We included 118 people with obesity who enrolled in a CLI at the outpatient clinic of the Obesity Center CGG (‘Centrum Gezond Gewicht’) at Erasmus MC University Medical Center, Rotterdam, The Netherlands. Each year, two groups of around 10 individuals each enroll in the 1.5 years program. A multidisciplinary team consisting of a dietician, a psychologist, and a physical therapist provides CLI group sessions that include nutritional advice, cognitive-behavioral therapy psychoeducation, and exercise sessions. A more detailed description of the intervention can be found elsewhere [47, 48].

Participants were included in the analysis based on the following criteria: age ≥ 18 years, BMI ≥ 30 kg/m^2^, and at least one obesity-related comorbidity (e.g., dyslipidemia, hypertension, non-alcoholic fatty liver disease, type 2 diabetes, obstructive sleep apnea, or osteoarthritis). Furthermore, exclusion criteria were other causes of obesity (e.g., genetic or endocrine diseases), inability to speak Dutch, intellectual disability (IQ < 80), current wish for pregnancy and severe physiological or behavioral problems impeding functioning in a group. Patients were excluded from follow-up analyses if they had developed medical conditions that interfered with the intervention, no treatment adherence (≥ three sessions missed), or were drop-outs. The data collection took place from October 2011 to April 2022.

### Measurements

Anthropometric measurements were performed at baseline (T0), after the first 10 weeks of intensive treatment (T1), and after 1.5 years at the end of the weight maintenance phase (T2). The participants’ body weight and WC were measured by trained outpatient clinic assistants. Body weight was measured in kilograms using a calibrated scale, with the patient wearing clothes and standing without shoes. BMI was calculated by dividing weight by height in meters squared (kg/m^2^). WC in centimeters was measured unclothed, halfway between the superior anterior iliac crest and the lowest rib after a normal expiration, and the average of two consecutive measurements was noted [47, 48].

#### Neighborhood Characteristics

In order to identify the neighborhood characteristics score, participants were asked to complete a 13-item questionnaire assessing neighborhood characteristics at baseline (see supplementary materials of this article). This questionnaire, to some extent including a selection of questions from previously used questionnaires, was developed in-house at the Erasmus MC Rotterdam, and measures neighborhood characteristics on different factors with conceptually related items. These factors included four items that, when combined, formed the factor neighborhood safety (e.g., “*The streets in my neighborhood are adequately lit in the evening and at night*”), four items that, when combined formed the factor neighborhood attractiveness (e.g., “*I find my neighborhood attractive to live in*”), three items that, when combined formed the factor social cohesion (e.g., “*The people in this neighborhood treat each other in a pleasant way*”), one item on access to grocery stores (“*I can do my daily shopping in my neighborhood*”), and one item on sport facilities (“*There are sports facilities in my neighborhood*”). Items were assessed on a 5-point Likert scale (1 = definitely agree to 5 = definitely disagree). The items of the questionnaire have a Cronbach’s alpha of 0.781 among the questions. Per factor, mean scores were determined, with higher scores indicating a perception of living in a neighborhood marked by less favorable characteristics. We reverse-coded the responses for questionnaire items a, b, h, and k (see supplementary material) to ensure that the scoring of these items aligns with that of the other questionnaire items, where higher scores reflect a less favorable neighborhood.

A sumscore was also computed by calculating the mean of all items together. This neighborhood characteristics total score reflects how individuals perceive their neighborhood, taking into account the conceptually related factors. The questionnaires were completed on paper or digital, depending on the year in which a participant completed the questionnaire.

#### Neighborhood Socioeconomic Status Score

Participants’ postal codes were assessed and linked to corresponding Dutch neighborhood socioeconomic status (NSES) scores. This was done by using NSES scores which Statistics Netherlands (CBS) publishes every 2 years. The NSES score provides a broader, general assessment of a neighborhood. More specifically these are NSES scores that focus on welfare (a combination of income and wealth), highest level of education, and recent labor participation of people living in a certain postal code number area [49, 50]. The NSES score of a neighborhood is the average of all total SES scores for the households in that neighborhood [50]. The NSES score used in the analysis corresponds to the year closest to the year the individual started the lifestyle intervention.

### Statistical Analysis

Multiple linear regression analyses were conducted to examine the relationship between the baseline assessment of neighborhood characteristics (independent variables) and the relative changes in BMI and WC (dependent variables), both from T0 to T1 (the initial weight loss), and from T0 to T2 (the long-term weight loss maintenance).

The relative change was calculated as ((new − old)/old × 100). The statistical analyses were performed on the sumscore of the thirteen items. Additionally, separate analyses were conducted on the specific characteristics, neighborhood unsafety, neighborhood unattractiveness, social cohesion, and access to grocery stores and sport facilities. Analyses were adjusted for age, sex (model 1), and educational level (model 2). Subsequently, analyses were adjusted for the NSES score (full model), since previous data has shown that failure to consider neighborhood-level confounding may result in severely biased associations [51].

A cross-sectional analysis and a longitudinal sensitivity analysis were conducted to account for the impact of COVID-19, by excluding participants who were affected by the pandemic, as COVID-19 significantly influenced people’s lifestyle and health. In the cross-sectional analysis, participants who enrolled after living under COVID-19 measures for at least 6 months were excluded. In the longitudinal analysis, individuals who participated during COVID-19 (more than 9 months) were excluded.

Statistical analyses were performed using the IBM SPSS Statistics software version 29. Statistical significance was set at *p* < 0.05. The study was approved by the medical ethical committee of Erasmus University Medical Center, Rotterdam, the Netherlands (MEC-2012-257).

## Results

### Baseline Characteristics

The participants (*n* = 118) had at baseline a mean age of 45 years (range, 18–68 years),a mean weight of 116 kg (standard deviation (SD) ± 19) and a mean BMI of 39 kg/m^2^ (SD ± 5). Table 1 provides an overview of the participants’ demographic characteristics, including sex and educational level.

**Table 1:** Patient characteristics and anthropometrics at baseline

**Variables**	**Baseline** (***n*** = 118)
**Sex (*n* female) (%)**	89 (75)
**Mean age in years (range)**	45 (18–68)
**Educational level (*n*, %)**	
Primary education	2 (1.7**)**
Secondary education	24 (20.5)
Vocational education	25 (21.4)
Higher education	66 (56.5)
**Neighborhood characteristics (mean ± SD)**	
**Neighborhood unsafety**	2.4 ± 0.8
**Neighborhood unattractiveness**	2.0 ± 0.7
**Less neighborhood social cohesion**	2.2 ± 0.8
**Less access to grocery sports**	1.7 ± 1.0
**Less access to sport facilities**	2.0 ± 1.0
**Neighborhood socioeconomic status score (mean ± SD)**	− 0.1 ± 0.3
**Anthropometrics (mean ± SD)**	
**BMI (kg/m**^**2**^**)**	39 ± 5
**Body weight (kg)**	116 ± 19
**WC (cm)**	115 ± 14

*BMI* body mass index (kg/m^2^), *WC* waist circumference, *SD* standard deviation

### Cross-Sectional Analysis

Analyses regarding neighborhood characteristics score and NSES score in relation to anthropometrics yielded varying results (Table 2). The linear regression analyses revealed no significant associations between any of the neighborhood characteristics and both BMI and WC at baseline (all *p* > 0.05). These findings remained unchanged after adjusting for potential confounding variables, including sex, age, educational level, and the NSES score (*p* > 0.05).

**Table 2:** Neighborhood characteristics, socioeconomic status, and BMI and WC at the start of the lifestyle intervention (*n* = 118)

Factors	Model 1				Model 2				Full model			
	**BMI**		**WC**		**BMI**		**WC**		**BMI**		**WC**	
	*β* (95%CI)	*p* value	*β* (95%CI)	*p* value	*β* (95%CI)	*p* value	*β* (95%CI)	*p* value	*β* (95%CI)	*p* value	*β* (95%CI)	*p* value
***Neighborhood characteristics score***												
**Neighborhood unsafety**	− .3 (− 1.5; .8)	.558	− .8 (− 2.0; 3.7)	.571	− .4 (− 1.5; .8)	.6	.8 (− 2.1; 3.6)	.601	− .5 (− 1.7; .7)	.452	.7 (− 2.2; 3.7)	.616
**Neighborhood unattractiveness**	.9 (− .4; 2.3)	.172	1.9 (− 1.4; 5.1)	.258	.9 (− .4; 2.3)	.165	1.9 (− 1.3; 5.2)	.239	.9 (− .5; 2.3)	.200	1.9 (− 1.4; 5.2)	.251
**Less social cohesion**	− .1 (− 1.4; 1.2)	.858	1.9 (− 1.1; 5.1)	.214	− .1 (− 1.4; 1.2)	.869	1.9 (− 1.1; 5.0)	.207	− .1 (− 1.4; 1.2)	.850	2.1 (− 1.0; 5.3)	.190
**Less access to grocery stores**	.4 (− .6; 1.3)	.432	1.0 (− 1.3; 3.3)	.372	.3 (− .6; 1.3)	.429	1.0 (− 1.2; 3.3)	.365	.4 (− .6; 1.3)	.463	1.0 (− 1.4; 3.3)	.424
**Less sport facilities**	.8 (− .1; 1.6)	.090	1.1 (− 1.0; 3.3)	.292	.8 (− 1.0; 1.7)	.078	1.3 (− .8; 3.5)	.299	.8 (− .1; 1.6)	.097	1.2 (− .9; 3.4)	.260
**Total score**	.6 (− 1.1; 2.4)	.481	3.4 (− .8; 7.7)	.109	.7 (− 1.1; 2.4)	.464	3.5 (− .7; 7.8)	.098	.5 (− 1.3; 2.3)	.602	3.6 (− .7; 7.9)	.101
***Socioeconomic status score***												
	.0 (− 3.2; 3.3)	.990	1.6 (− 6.4; 9.5)	.696								

Model 1 is adjusted for sex and age. Model 2 is adjusted for sex, age, and educational level. The full model is adjusted for sex, age, educational level, and the neighborhood socioeconomic status score

*BMI* body mass index (kg/m^2^), *WC* waist circumference, *CI* confidence interval

### Longitudinal Analysis

Of the 118 patients for whom baseline data were available, 12 were excluded for the baseline (T0) to 10 weeks (T1) analysis, due to developing medical conditions that interfered with the intervention (*n* = 4), no treatment adherence (missed > 3 sessions) (*n* = 4), or no available anthropometrics (*n* = 4). Of these 106 patients of whom T0-T1 data was available, 31 were excluded for the T0-end of program at 1.5 years (T2) analysis, due to developing medical conditions that interfered with the intervention (*n* = 9), bariatric surgery (*n* = 1), no treatment adherence (missed > 3 sessions) (*n* = 2), no endpoint parameters measured (*n* = 8), and drop-out (*n* = 11). Thus, a total of 75 patients were included for T0-T2 analysis.

In the group of 75 people that were included on all time points, people lost on average 4.9 kg (4.1%) weight and 5.1 cm (4.3%) in WC between T0 and T1. Similarly, between T0 and T2, people lost 5.2 kg (4.4%) weight and 5.0 cm (4.3%) in WC.

#### Neighborhood Characteristics Score

Analyses yielded varying results (see Table 3). There was a statistically significant association between the perception of more neighborhood unsafety and a greater relative increase in BMI from T0 to T1 (*β* = 1.1 [95%CI 0.4; 1.8, *p* = 0.004 in the full model), thereby indicating less BMI improvement from T0 to T1.

**Table 3:** Neighborhood characteristics, neighborhood socioeconomic status, and relative changes in BMI and WC in response to a lifestyle intervention

			**Model 1**				**Model 2**				**Full model**				
			**%∆ BMI**		**%∆ WC**		**%∆ BMI**		**%∆ WC**		**%∆ BMI**		**%∆ WC**		
**Factors**	Period	*n*	*β* (95%CI)	*p* value	*β* (95%CI)	*p* value	*β* (95%CI)	*p* value	*β* (95%CI)	*p* value	*β* (95%CI)	*p* value	*β* (95%CI)	*p* value	
***Neighborhood characteristics score***															
**Neighborhood unsafety**	T0-T1	104	**1.3** **(.6; 2.1)**	< .001***	.9 (− .4; 2.2)	.155	**1.3** **(.6; 2.0)**	< .001***	.9 (− .4; 2.1)	.162	**1.1** **(.4; 1.8)**	.004**	.6 (− .6; 1.9)	.327	
	T0-T2	75	**3.2** **(1.3; 5.2)**	.002**	**2.7** **(.5; 4.9)**	.015*	**3.3** **(1.2; 5.1)**	.002**	**2.7** **(.5; 4.9)**	.015*	**3.1** **(1.1; 5.1)**	.003**	**2.8** **(.6; 5.1)**	.014*	
**Neighborhood unattractiveness**	T0-T1	103	− .4 (− 1.3; .5)	.385	.4 (− 1.1; 1.9)	.571	− .4 (− 1.3; .5)	.381	.5 (− .9; 2.0)	.512	− .6 (− 1.5; .3)	.173	.3 (− 1.2; 1.7)	.722	
	T0-T2	74	1.6 (− .6; 3.8)	.158	1.8 (− .6; 4.2)	.148	1.7 (− .6; 3.9)	.139	1.8 (− .6; 4.3)	.138	1.6 (− .7; 3.8)	.168	1.9 (− .6; 4.3)	.136	
**Less social cohesion**	T0-T1	104	.6 (− .3; 1.4)	.174	1.3 (− .0; 2.6)	.057	.6 (− .3; 1.4)	.176	1.3 (− .1; 2.6)	.060	.4 (− .4; 1.2)	.377	1.1 (− .3; 2.4)	.117	
	T0-T2	74	1.0 (− 1.1; 3.2)	.342	.7 (− 1.6; 3.1)	.518	1.0 (− 1.1; 3.2)	.354	.8 (− 1.6; 3.1)	.526	.9 (− 1.3; 3.0)	.424	.8 (− 1.6; 3.2)	.520	
**Less access to grocery stores**	T0-T1	104	− .3 (− .9; .4)	.429	− .2 (− 1.2; .8)	.690	− .3 (− .9; .4)	.430	− .2 (− 1.2; .8)	.714	− .1 (− .7; .5)	.812	.0 (− 1.0; 1.0)	.993	
	T0-T2	74	− .1 (1.8; 1.6)	.932	1.2 (− .6; 3.0)	.200	− .2 (1.9; 1.5)	.825	1.1 (− .7; 3.0)	.234	− .1 (− 1.8; 1.6)	.905	1.1 (− .8; 3.0)	.239	
**Less sport facilities**	T0-T1	104	.2 (− .4; .8)	.483	.3 (− .7; 1.2)	.587	.2 (− .4; .8)	.485	.4 (− .6; 1.3)	.455	.2 (− .4; .8)	.511	.3 (− .6; 1.2)	.514	
	T0-T2	74	.5 (− 1.0; 2.1)	.483	.8 (− .8; 2.5)	.331	.5 (− 1.0; 2.1)	.488	.8 (− .9; 2.5)	.334	.5 (− 1.0; 2.0)	.507	.8 (− .9; 2.5)	.336	
**Less sport facilities and less access to grocery stores combined**	T0-T1	104	− .0 (− .7; .7)	.986	.1 (− 1.0; 1.2)	.922	− .0 (− .7; .7)	.982	.1 (− 1.0; 1.3)	.817	.1 (− .6; .8)	.797	.2 (− .9; 1.3)	.701	
	T0-T2	74	.4 (− 1.5; 2.2)	.707	1.3 (− .7; 3.3)	.197	.3 (− 1.6; 2.2)	.767	1.3 (− .8; 3.3)	.218	.3 (− 1.6; 2.2)	.738	1.3 (− .8; 3.3)	.222	
**Total score**	T0-T1	103	.9 (− .2; 2.1)	.120	1.4 (− .5; 3.3)	.152	.9 (− .3; 2.1)	.124	1.5 (− .4; 3.4)	.131	.6 (− .6; 1.7)	.322	1.1 (− .8; 3.0)	.265	
	T0-T2	75	**3.3** **(.5; 6.2)**	.021*	**3.3** **(.3; 6.4)**	.033*	**3.3** **(.5; 6.2)**	.022*	**3.3** **(.3; 6.4)**	.034*	**3.2** **(.3; 6.1)**	.031*	**3.4** **(.3; 6.6)**	.032*	
***Socioeconomic status score***															
	T0-T1	106	− **3.4** **(**− **5.4;** − **1.4)**	.001***	− **3.7** **(**− **7.1;** − **.3)**	.034*									
	T0-T2	75	− 3.4 (− 9.4; 2.5)	.255	.487 (− 6.1; 7.1)	.883									

Model 1 is adjusted for sex and age. Model 2 is adjusted for sex, age, and educational level. The full model is adjusted for sex, age, educational level, and the socioeconomic status score

*BMI* body mass index (kg/m^2^), *WC* waist circumference, *CI* confidence interval

p < 0.001 is ***, p < 0.01 is **, and p < 0.05 is *

No significant associations were found in neighborhood unattractiveness, less social cohesion, and less access to grocery stores and sport facilities with relative changes in BMI and WC (*p* > 0.05). With regard to less social cohesion, model 1 and model 2 showed a non-significant trend between higher scores on perceived less social cohesion and greater relative increase in WC at T0-T1 (*β* = 1.3 [95%CI − 0.1; 2.6], *p* = 0.060 in model 2), thereby indicating less WC improvement from T0 to T1, which disappeared between T1 and T2 (Table 3).

Furthermore, a significant association was found between more perceived neighborhood unsafety and both greater relative increase in BMI and WC from T0-T2, indicating less BMI and WC improvement from T0 to T2 (*β* = 3.1 [95%CI 1.1; 5.1, *p* = 0.003; *β* = 2.8 [95%CI 0.6; 5.1], *p* = 0.014, respectively, in the full models). Significant associations were also observed between the total score of neighborhood factors and changes in BMI and WC. After the multivariable adjustment, a higher total score of perceived unfavorable neighborhood characteristics was on the long term associated with greater relative increase in BMI and WC, thereby indicating less improvement in BMI and WC (*β* = 3.2 [95%CI 0.3; 6.1], *p* = 0.031; *β* = 3.4 [95%CI 0.3; 6.6], *p* = 0.032, respectively, in the full model).

#### Neighborhood Socioeconomic Status Score

The results show a significant association between higher baseline socioeconomic status scores and a greater relative decrease in BMI from T0 to T1 (*β* =  − 3.4 [95%CI − 5.4; 1.4], *p* = 0.001) as well as greater relative decrease in WC (*β* =  − 3.7 [95%CI − 7.1; − 0.3], *p* = 0.034). However, after 1.5 years, this association was no longer statistically significant (*p* > 0.05). Table 3 provides information on the relative changes in BMI and WC in relation to the socioeconomic status scores.

### Sensitivity Analysis

In a sensitivity analysis accounting for the impact of COVID-19, the relationship between the neighborhood characteristics and the anthropometric measurements was assessed. Results from the sensitivity analysis, excluding patients from the COVID-19 period, did not differ considerably from the larger group. Details are displayed in Supplementary Tables S1 and S2 Due to the small sample size, it was not possible to account for NSES score in these analyses.

In this group of 49 people that were still included at T2, people on average lost 5.7 kg (4.6%) weight and 6.1 cm (5.1%) in WC between T0 and T1. Similarly, between T0 and T2, people lost 6.2 kg (5%) weight and 5.8 cm (4.8%) in WC.

## Discussion

This study shows that individuals with obesity living in less favorable neighborhoods may have greater challenges regarding BMI and WC reduction following a 1.5-year multidisciplinary combined lifestyle intervention with cognitive-behavioral therapy.

Previous research has shown that living in an unsafe neighborhood was associated with a greater probability of obesity [44]. It has also been shown that people who report that there was too much crime in their neighborhood to go outside for walks or play during the day were more likely to live with overweight or obesity [52]. In addition, neighborhood crime is longitudinally associated with greater body mass index [53] and neighborhood crime measured by the homicide rate is significantly associated with less weight loss [54]. These findings align with the results of the present study, wherein it was observed, that neighborhood unsafety plays an important role in determining the success of a lifestyle intervention, both in the short and long term.

Furthermore, previous research indicates the importance of the social environment. A great breadth of literature describes the impact of the social environment on weight loss and the prevalence of overweight and obesity [13, 28]. Where one study finds a link between high social capital and better health [43], another finds that living in a non-supportive neighborhood was associated with a higher probability of living with overweight and obesity when compared with their peers living in supportive neighborhoods [44]. Based on this previous research, it was hypothesized that social cohesion might contribute to the success of a lifestyle intervention. However, in the present study, regarding social cohesion, compelling evidence was not found. Nevertheless, a noticeable trend was observed: although not statistically significant, there was a trend towards an association between higher scores in perceived less social cohesion and less short-term improvements in WC, following the combined lifestyle intervention.

In addition to examining neighborhood safety and social cohesion, this study investigated other neighborhood characteristics. Interestingly, within the conceptually related factors of neighborhood attractiveness and access to sports facilities or grocery stores, we did not find any significant associations with changes in anthropometrics during the lifestyle intervention. This is noteworthy given previous research demonstrating the importance of various other characteristics, such as the effects of disparities in access to supermarkets and fast-food outlets on overweight [20, 22, 34, 36, 37, 41]. It may be possible that other unmeasured factors, such as individual dietary habits or physical activity levels, play a more prominent role in determining changes in anthropometrics in this context.

Moreover, a higher neighborhood characteristics total score, indicating a perception of a less favorable neighborhood, was significantly associated with less relative long-term reductions in WC and BMI, following the combined lifestyle intervention. These findings persisted even after adjusting for the NSES score. These observations raise the question how much importance the safety score holds within the total score. In the data, we observed significant positive correlations between the four factors of neighborhood characteristics, without detecting multicollinearity. Given that prior research indicates that environmental factors (similar to those used in this current study) contribute to the prevalence of overweight and obesity, with underlying mechanisms such as outdoor physical activity, it stands to reason that all factors contribute to the significance of the total score. One could speculate that the conceptually related items measured might be interconnected, and thus, that the true impact only becomes visible when considering them cumulatively [16, 18, 19]. This notion aligns with research examining, for instance, the role of neighborhood deprivation as a key determinant of childhood obesity [21].

Although this study did not delve into the specific underlying explanations of how neighborhood characteristics impact lifestyle interventions, it is likely that the same mechanisms operate through which overweight and obesity become more prevalent among persons with initially healthy body weight. These include reduced outdoor physical activity in unsafe neighborhoods [55], lower neighborhood walkability [56], and the impact of neighborhood characteristics on stress levels [54, 57–61]. Stress levels influenced by neighborhood features can contribute to unhealthy eating habits and weight gain [62]. The study’s findings suggest that limited social cohesion may hinder the effectiveness of lifestyle interventions, potentially restricting weight loss in the initial 10 weeks. Social cohesion typically fosters support and motivation, enhancing adherence to healthy behaviors and intervention outcomes [14, 33, 54, 63, 64]. Possibly, factors such as less opportunities for community engagement and other competing priorities could further exacerbate the challenges faced in implementing lifestyle interventions within certain neighborhoods.

To account for the impact of COVID-19, a sensitivity analysis was conducted. Neighborhood characteristics did not differ considerably from the larger group. However, when excluding people who participated during the COVID-19 period, the effect of the lifestyle intervention on BMI and WC loss is greater, which is in line with previous studies on this lifestyle intervention, with a mean weight loss off 5.15% and a decrease in waist circumference of 6.41% after 1.5 years [47, 48].

### Strengths and Limitations

The strengths of the present study lie in its longitudinal approach, our ability to link self-reported neighborhood characteristics to the changes in objectively measured BMI and WC, and the stringent control for socioeconomic factors at both the individual and neighborhood levels.

The study has several limitations that merit acknowledgement. Firstly, the questionnaire used to assess neighborhood characteristics lacked formal validation. However, it is noteworthy that the items of the questionnaire exhibit an acceptable internal consistency with a Cronbach’s alpha of 0.781 among the questions. Another limitation to consider is the small sample size utilized in this study, which is a common concern in social sciences. Linear regression analysis based on a sample of 118 individuals may not guarantee the robustness of the analysis results and further research in larger samples is needed. Additionally, the testing of numerous relationships in the study may increase the risk of type 1 error. Despite this consideration and the exploratory nature of this study, the results are suggestive and valuable for future research.

### Implications for Practice and Considerations for Future Studies

Our results imply that addressing the overall quality of neighborhoods could be a strategic approach to facilitate weight management and encourage healthier lifestyle behaviors among the population. By promoting more favorable, safer neighborhoods with supportive neighborhood environments, public health interventions have the potential to enhance the effectiveness and impact of efforts to combat obesity and its associated health risks on a community level. Furthermore, professionals working in lifestyle interventions might need to have extra attention for participants of lifestyle programs residing in less favorable neighborhoods. Possibly, professionals could provide guidance to enhance physical activity opportunities within their local surroundings and offer strategies for individuals who perceive safety concerns, enabling them to engage in outdoor activities with a greater sense of security. Combined lifestyle interventions need not be seen as one-size-fits-all but should be adapted to local living circumstances.

This study may not find some associations due to its smaller sample size or the relatively smaller variations within the study group for these specific factors; therefore, conducting similar research on a larger sample size would be interesting. Furthermore, this study indicates that the social and physical environment people live in may play a role in the success of the intervention. We, however, need more research to study whether this is causally related. Lastly, more research is needed to better understand the complex interplay between neighborhood characteristics and health outcomes, taking into account a broader range of confounders, such as stress.

## Conclusion

The findings suggest that individuals with higher scores in living in perceived less favorable neighborhoods may experience less favorable outcomes in terms of BMI and waist circumference reduction following a lifestyle intervention, irrespective of the socioeconomic status score of the neighborhood one lives in. Given the limitations of the study, caution should be exercised when drawing broad conclusions. Nevertheless, these results suggest a potential connection between neighborhood perceptions and the effects of a lifestyle intervention. Further studies are needed to confirm these associations and to assess whether this is related to increased stress levels or other environmental, social, or neighborhood factors.

## Supplementary Information

Below is the link to the electronic supplementary material.

Supplementary file1 (DOCX 24 KB)

## Data Availability

The dataset presented in the study is available on request from the corresponding author. The data are not publicly available due to privacy issues.
