# Supplementary material for: Neighborhood Characteristics Related to Changes in Anthropometrics During a Lifestyle Intervention for Persons with Obesity
Source: Int J Behav Med. 2024 Sep 11;32(1):58–68. doi: 10.1007/s12529-024-10317-y (PMC11790805; doi:10.1007/s12529-024-10317-y)
Supplement: Supplementary file 1 — Supplementary file1 (DOCX 24 KB) [file 12529_2024_10317_MOESM1_ESM.docx]

**Supplementary materials:**

| **Supplementary table S1.** Sensitivity analysis: neighborhood characteristics, socioeconomic status and BMI and WC at the start of the lifestyle intervention, with exclusion of individuals who enrolled after living in 6 months of COVID-19 measures (n=89) | | | | | | | | | | | | |
| --- | --- | --- | --- | --- | --- | --- | --- | --- | --- | --- | --- | --- |
|  | **Model 1** | | | | **Model 2** | | | | **Full model** | | | |
|  | **BMI** | | **WC** | | **BMI** | | **WC** | | **BMI** | | **WC** | |
| **Factors** | β (95%CI) | p value | β (95%CI) | p value | β (95%CI) | p value | β (95%CI) | p value | β (95%CI) | p value | β (95%CI) | p value |
| ***Neighborhood characteristics score*** | | | | | | | | | | | | |
| **Neighborhood unsafety** | -.694 (-2.191 ; .804) | .360 | -1.011 (-4.622 ; 2.601) | .579 | -.697 (-2.205 ; .811 | .361 | -.955 (-4.629 ; 2.640) | .588 | -.731 (-2.314 ; .852) | .361 | -.599 (-4.404 ; 3.205) | .755 |
| **Neighborhood unattractiveness** | .815 (-.676 ; 2.305) | .280 | 2.164 (-1.379 ; 5.706) | .288 | .814 (-.685 ; 2.314) | .283 | 2.154 (-1.406 ; 5.714) | .232 | .853 (-.672 ; 2.378) | .269 | 2.464 (-1.128 ; 6.057) | .176 |
| **Less social cohesion** | -.104 (-1.545 ; 1.337) | .886 | 1.672 (-1.773 ; 5.118) | .337 | -.102 (-1.553 ; 1.348) | .889 | 1.658 (-1.809 ; 5.126) | .344 | -.089 (-1.573 ; 1.394) | .905 | 1.992 (-1.553 ; 5.516) | .264 |
| **Less access to grocery stores** | .153 (-.891 ; 1.196) | .772 | 1.054 (-1.445 ; 3.553) | .404 | .153 (-.897 ; 1.203) | .773 | 1.051 (-1.463 ; 3.564) | .408 | .147 (-.925 ; 1.218) | .786 | .896 (-1.661 ; 3.453 | .488 |
| **Less sport facilities** | .346 (-.661 ; 1.353) | .497 | .473 (-1.952 ; 2.898) | .699 | .346 (-.667 ; 1.369) | .503 | .526 (-1.936 ; 2.988) | .672 | .349 (-.681 ; 1.378) | .240 | .564 (-1.904 ; 3.031) | .651 |
| **Total score** | .311 (-1.760 ; 2.381) | .766 | 2.464 (-2.439 ; 7.367) | .320 | .312 (-1.722 ; 2.396) | .766 | 2.510 (-2.419 ; 7.439) | .314 | .369 (-1.798 ; 2.537) | .736 | 3.242 (-1.810 ; 8.295) | .205 |
| ***Socioeconomic status score*** | | | | | | | | | | | | |
|  | .326 (-3.569 ; 4.222) | .868 | 4.450 (-4.937 ; 13.837) | .349 |  |  |  |  |  |  |  |  |

_Model 1 is adjusted for sex and age. Model 2 is adjusted for sex, age and educational level. The full model is adjusted for sex, age, educational level and the socioeconomic status score_

_Abbreviations: BMI, body mass index (kg/m2); WC, waist circumference; CI, confidence interval_

**Supplementary table S2.** Sensitivity analysis: neighborhood characteristics, neighborhood socioeconomic status and relative changes in BMI and WC in response to lifestyle intervention, excluding people who participated during COVID-19 (more than 9 months)

|  | |  |  |  |  |  |  |  |  |  |  |  |  |  |  |  |
| --- | --- | --- | --- | --- | --- | --- | --- | --- | --- | --- | --- | --- | --- | --- | --- | --- |
|  | | | |  |  |  | **Model 1** | | | | | | **Model 2** | | | |
|  | | | |  |  | **%∆ BMI** | | |  | **%∆ WC** | | | **%∆ BMI** | | **%∆ WC** | |
| **Factors** | | | | Period | n | β (95%CI) | | p value | β (95%CI) | |  | p value | β (95%CI) | p value | β (95%CI) | p value |
|  | ***Neighborhood characteristics score*** | | | | | | | | | | | | | | | |
| **Neighborhood unsafety** | | | | T0-T1 | 73 | **1.267**  **(.212 ; 2.323)** | | **.019*** | 1.174  (-.576 ; 2.923) | |  | .185 | **1.253  (.186 ; 2.319)** | **.022*** | 1.231  (-.515 ; 2.976) | .164 |
|  |  |  |  | T0-T2 | 48 | **3.401**  **(.637 ; 6.165)** | | **.017*** | 1.951  (-1.254 ; 5.156) | |  | .226 | **3.586  (.797 ; 6.374)** | **.013*** | 2.239  (-.962 ; 5.441) | .166 |
| **Neighborhood unattractiveness** | | | | T0-T1 | 70 | -.241  (-1.387 ; .904) | | .675 | 1.002  (-.749 ; 2.753) | |  | .257 | -.276  (-1.430 ; .879) | .635 | 1.077  (-.677 ; 2.832) | .224 |
|  |  |  |  | T0-T2 | 48 | 1.378  (-1.156 ; 3.912) | | .279 | .870  (-1.955 ; 3.695) | |  | .538 | 1.400  (-1.152 ; 3.953) | .275 | .913  (-1.900 ; 3.727) | .516 |
| **Less social cohesion** | | | | T0-T1 | 71 | .530  (-.627 ; 1.687) | | .364 | 1.344  (-.467 ; 3.154) | |  | .143 | .567  (-.603 ; 1.736) | .337 | 1.228  (-.598 ; 3.055) | .184 |
|  |  |  |  | T0-T2 | 48 | .337  (-2.514 ; 3.188) | | .813 | -.144  (-3.295 ; 3.008) | |  | .927 | .269  (-2.613 ; 3.151) | .851 | -.286  (-3.433 ; 2.861) | .855 |
| **Less access to grocery stores** | | | | T0-T1 | 71 | -.095  ( -.832 ; .624) | | .798 | -.127  (-1.264 ; 1.010) | |  | .824 | -.091  (-.832 ; .650 | .808 | -.146  (-1.280 ; .989) | .798 |
|  |  |  |  | T0-T2 | 48 | .928  (-.908 ; 2.763) | | .314 | 1.500  (-.500 ; 3.500) | |  | .138 | .832  (-1.086 ; 2.749) | .387 | 1.278  (-.798 ; 3.354) | .221 |
| **Less sport facilities** | | | | T0-T1 | 71 | .383  (-.308 ; 1.073) | | .273 | .470  (-.598 ; 1.538) | |  | .383 | .363  (-.341 ; 1.068) | .307 | .585  (-.492 ; 1.661) | .282 |
|  |  |  |  | T0-T2 | 48 | 1.433  (-.299 ; 3.166) | | .103 | 1.575  (-.339 ; 3.490 | |  | .104 | 1.427  (-.319 ; 3.173) | .107 | 1.562  (-.344 ; 3.468) | .106 |
| **Total score** | | | | T0-T1 | 73 | .790  (-.700 ; 2.281) | | .293 | 1.639  (-.693 ; 3.971) | |  | .165 | .753  (-.751 ; 2.256) | .321 | 1.723  (-.611 ; 4.057) | .145 |
|  |  |  |  | T0-T2 | 49 | 2.934 (-.379 ; 6.247) | | .081 | 2.082  (-1.655 ; 5.818) | |  | .268 | 2.932  (-.405 ; 6.269) | .083 | 2.078  (-1.642 ; 5.799) | .266 |
|  | ***Socioeconomic status score*** | | | | | | | | | | | | | | | |
|  | | | | T0-T1 | 73 | **-2.940 (-5.691 ; -.189)** | | **.037*** | -4.086 (-8.363 ; .191) | |  | .061 |  |  |  |  |
|  |  |  |  | T0-T2 | 49 | 2.559  (-5.070 ; 10.188) | | .503 | 3.626  (-4.774 ; 12.026) | |  | .389 |  |  |  |  |

_Model 1 is adjusted for sex and age. Model 2 is adjusted for sex, age and educational level
P < 0.001 is ***, p < 0.01 is ** and p < 0.05 is *, Abbreviations: BMI, body mass index (kg/m2); WC, waist circumference; CI, confidence interval_

**Supplementary table S3.** Coefficients of univariate linear models regarding sex, age, educational level and the socioeconomic status score, regarding the prediction of baseline BMI and WC as well as T0-T1 and T0-T2 changes in BMI and WC

| Coefficients of covariates | **n** | **β (95%CI)** | **p value** |
| --- | --- | --- | --- |
| ***T0*** | 118 |  |  |
| **Sex** |  | 1.149 (-.991; 3.289) | .290 |
| **Age** |  | -.059 (-.134; .015) | .116 |
| **Educational level** |  | .141(-.506; .788) | .667 |
| **Socioeconomic status score** |  | .161 (-3.116; 3.348) | .923 |
| ***T0 – T1*** | 106 |  |  |
| **Sex** |  | .946 (-.463; 2.356) | .186 |
| **Age** |  | -.019 (-.068; .030) | .441 |
| **Educational level** |  | -.019 (-.445; .407) | .929 |
| **Socioeconomic status score** |  | -3.304 (-5.351; -1.256) | .002 |
| ***T1 – T2*** | 75 |  |  |
| **Sex** |  | 1.352 (-2.204; 4.908) | .451 |
| **Age** |  | -.009 (-.135; .116) | .884 |
| **Educational level** |  | .560 (-.538; 1.658) | .313 |
| **Socioeconomic status score** |  | -3.530 (-9.92; 2.232) | .226 |

**Supplementary**

Questionnaire concerning neighborhood

Below are several statements about your neighborhood. By "neighborhood," we mean the area around your home that you can reach within a 10-minute walk. Can you indicate whether you agree or disagree with the following statements?

Definitely agree

Agree

Neither agree nor disagree

Disagree

Definitely disagree

***Questionnaire items***

1. I am sometimes afraid of crime or being harassed when walking through my neighborhood.
2. When I walk or cycle through my neighborhood, I sometimes feel unsafe due to the busy traffic.
3. The streets in my neighborhood are sufficiently illuminated during evenings and nights.
4. There are trees along the road in my neighborhood.
5. There is a small park in my neighborhood.
6. My neighborhood looks tidy.
7. I find my neighborhood attractive to live in.
8. There are sometimes groups of youths hanging around in my neighborhood.
9. I can do daily groceries in my neighborhood.
10. There are sports facilities in my neighborhood.
11. I often feel lonely in this neighborhood.
12. People in this neighborhood interact with each other in a pleasant manner.
13. People in this neighborhood are willing to help each other.

(Un)safety: items a, b, c, h
(Un)attractiveness: items d, e, f, g

(Less) access to grocery stores and sport facilities: i, j

(Less) social cohesion: k, l, m
